# Learning the Quantum Centroid Force Correction in Molecular Systems: A Localized Approach

**DOI:** 10.3389/fmolb.2022.851311

**Published:** 2022-05-19

**Authors:** Chuixiong Wu, Ruye Li, Kuang Yu

**Affiliations:** Tsinghua-Berkeley Shenzhen Institute, Tsinghua Shenzhen International Graduate School, Tsinghua University, Shenzhen, China

**Keywords:** molecular dynamics, machine learning, nuclear quantum effects, path-integral molecular dynamics, centroid molecular dynamics

## Abstract

Molecular mechanics (MM) is a powerful tool to study the properties of molecular systems in the fields of biology and materials science. With the development of ab initio force field and the application of ab initio potential energy surface, the nuclear quantum effect (NQE) is becoming increasingly important for the robustness of the simulation. However, the state-of-the-art path-integral molecular dynamics simulation, which incorporates NQE in MM, is still too expensive to conduct for most biological and material systems. In this work, we analyze the locality of NQE, using both analytical and numerical approaches, and conclude that NQE is an extremely localized phenomenon in nonreactive molecular systems. Therefore, we can use localized machine learning (ML) models to predict quantum force corrections both accurately and efficiently. Using liquid water as example, we show that the ML facilitated centroid MD can reproduce the NQEs in both the thermodynamical and the dynamical properties, with a minimal increase in computational time compared to classical molecular dynamics. This simple approach thus largely decreases the computational cost of quantum simulations, making it really accessible to the studies of large-scale molecular systems.

## Introduction

Molecular mechanics (MM) simulation is an extremely powerful tool in the studies of biomolecules and material systems. In most situations, MM simulations are conducted with Born-Oppenheimer approximation, with the atoms moving on an adiabatic electronic potential energy surface (PES). The motions of the atoms on PES are typically governed by classical statistical mechanics and Newtonian dynamics, so the nuclear quantum effects (NQEs) are often neglected. While many properties can be computed with satisfactory accuracy at classical level, it has been shown that the NQE can be quite essential in systems with light atoms (Wang et al., 2014; Markland and Ceriotti, 2018; Tuckerman and Ceperley, 2018). One important example is that NQE may significantly alter the strengths and the structures of the hydrogen bonding networks in aqueous solutions. Such effect brings profound impacts to the understandings of many important biological problems such as enzyme activities and DNA stabilities (Agarwal et al., 2002; Pérez et al., 2010). In materials science, it was also found that NQEs play nonnegligible roles in many problems such as guest molecule adsorptions and thermal transport simulations (Wahiduzzaman et al., 2014; Shulumba et al., 2017; Luo and Yu, 2020). Existing evidences show that NQE is not only a specific problem existing in a small number of systems, but a universal phenomenon that needs to be accounted for in most biological and materials simulations.

Conventionally, NQEs can be partially incorporated into classical MM through implicit ways. Ad hoc corrections can be used for some systems: for example, water molecules are often constrained to be rigid, simulating the freezing of the high-frequency intramolecular vibrations at room temperature. However, such techniques are highly specific to a certain type of molecule and can lead to significant errors as it does not treat all degrees of freedom consistently (Luo and Yu, 2020) Meanwhile, the force fields most widely used nowadays in molecular simulations (such as AMBER (Weiner et al., 1984), OPLS (Jorgensen et al., 1984, 1996), and CHARMM (Brooks and Karplus, 1983; MacKerell et al., 1995; MacKerell Jr et al., 2000) were developed empirically to reproduce the experimental results. Since the real world is quantum in nature, it can be argued that these empirical force field parameters are trained with quantum effects incorporated implicitly, legitimating their usages in classical MM simulations. However, we note that in the conventional force field training process, it is often difficult to fit both the small cluster ab initio data and the bulk experimental data simultaneously. Therefore, in order to obtain a balanced performance in bulk, one often needs to sacrifice the accuracy of the potential in the small clusters. Besides the long-range many-body interactions, the negligence of NQE in bulk simulations is potentially one of the major reasons causing this dilemma. Due to this problem, the conventional empirical force fields are often not reliable in the details of the PES, which limits its transferability and its predictive power. Therefore, in the past decade, it has becoming increasingly popular to construct accurate force fields from ab initio data directly without any experimental inputs (Lee et al., 1995; Martin et al., 1995, 95; McDaniel et al., 2012; Xu et al., 2018). For the systems that are affordable, ab initio MD (AIMD), including both Born-Oppenheimer MD (BOMD) and Car-Parinello MD (CPMD) (Car and Parrinello, 1985), are also frequently performed with the ab initio calculations being run on the fly to compute the PES. For these nonempirical MM simulations, NQE becomes an important issue with universal concerns and should be addressed explicitly in the simulation.

Most main stream NQE methods that are widely used in large-scale simulations are originated from Feynman’s path-integral formula (Feynman, 1998; Feynman et al., 2010). One simple technique is the Feynman-Hibbs correction (FHC) (Sesé, 1992, 1995; Guillot and Guissani, 1998; Feynman et al., 2010), in which atoms are smeared as in free-particle limit and the quantum correction is given by convoluting the PES with Gaussian distributions. In a pairwise additive potential, FHC also leads to a pairwise additive correction, which are only related to the high order derivatives of the pairwise interactions. Although being heavily approximated, FHC has been widely used in complicated simulations (e.g., Grand Canonical Monte Carlo simulations (Kowalczyk et al., 2005; Fischer et al., 2009; Durette et al., 2016; Ahmed et al., 2017, 2019)) due to its simplicity. However, FHC can be erroneous in many systems (as we will show in this work), and it can be difficult to implement for a general many-body potential. A more advanced method is the path-integral molecular dynamics (PIMD) (Berne and Thirumalai, 1986; Tuckerman and Martyna, 2000), which simulates a quantum particle using a classical “ring-polymer,” realizing the mathematical connection between the partition functions of the two systems. In order to describe the dynamics, PIMD is further succeeded by techniques such as ring-polymer molecular dynamics (RPMD) (Craig and Manolopoulos, 2004) and centroid molecular dynamics (CMD) (Cao and Voth, 1994a; 1994b; 1994c; 1994d; 1994e). All these methods can generate the exact quantum Boltzmann distribution when using large number of beads, but they are also much more expensive to run compared to classical MD. The large computational cost of PIMD roots in two facts: first, it utilizes multiple (typically 32–64) beads to represent one atom, so the number of energy and force evaluations it needs is much more than that of a classical simulation; and second, the hard spring potential between the neighboring beads requires a much smaller timestep for time integration. Tremendous efforts have been made to accelerate PIMD (including RPMD and CMD): for example, algorithms such as contraction (Markland and Manolopoulos, 2008b, 08, 2008a) or high-order PI(Pérez and Tuckerman, 2011; Kapil et al., 2016) reduces the number of beads effectively, and advance integrators were developed to allow the use of larger timesteps (Ceriotti et al., 2010). These efforts have made PIMD gradually enter the mainstream of MD simulations, but it is still much slower than the standard classical MD. Techniques such as contraction also relies on a subjective partition of the potential into fast and slow varying parts, which is sometimes not so straightforward to perform. Consequently, it is still relatively rare to see PIMD (and the related RPMD/CMD methods) being used on large systems (e.g., in biomolecule simulations) in conjunction with expensive many-body potentials (e.g., multipolar polarizable force fields, or even ab initio potentials).

In this work, using water as example, we will demonstrate how machine learning (ML) techniques can be utilized to accelerate the CMD simulation. CMD is an important category of path-integral method, which essentially coarse grains each ring-polymer into a single site located on the centroid of the polymer. The centroids move on an effective potential of mean force (PMF), which are conventionally sampled on-the-fly using explicit ring-polymers. The masses of the intra-polymer modes are set to be light, so the centroid and the intra-polymer motions are decoupled and the centroid motions can be considered as adiabatic. Rigorous adiabatic CMD usually requires a much smaller timestep, and the computation of the centroid PMF is also time consuming. However, noticing that the difference between the centroid PMF and the original PES (i.e., the quantum correction to the PES) is localized, it can be learned using localized ML models quite efficiently.

In the recent few years, we witness a rapid development of ML techniques in MM simulations. A variety of ML techniques, including BPNN (Behler and Parrinello, 2007), EANN (Zhang et al., 2019, 2021), SchNet (Schütt et al., 2018, 18), and DeepPotential (Zhang et al., 2018, 18), have been applied to develop accurate high-dimensional PES. All these models decompose the total energy into a sum of localized atomic energies, which are then predicted using neural networks with input features designed to describe the local environment of each atom. While such approaches have become increasingly popular in force field development, their applications on the description of NQE are relatively rare. Liu et al. developed the method called equilibrium continuity dynamics with the aim of calculating the quantum time correlation function in Wigner phase space, and accelerated this approach using ML techniques (Liu et al., 2021). However, to the best of our knowledge, the ML-facilitated path-integral methods (in particular, CMD) have not been thoroughly investigated. In this work, through analytical and numerical methods, we will rigorously quantify the locality of the CMD quantum force correction in molecular systems. Due to this locality, the CMD quantum force correction is actually much easier to model using ML methods compared to regular PES. We will show that a robust quantum force correction can be trained efficiently on very small cluster PIMD samples in molecular systems. Using this method, we can perform CMD simulations with a speed similar to the conventional classical MD, really making the quantum simulations accessible to the studies of large-scale biological and materials problems.

## Methods

### Path-Integral Based Methods

According to Feynman, the quantum partition function of a particle can be expressed in a path integral form, which, after discretization, is analogous to the classical partition function of a ring-polymer. For example, in the one-dimensional case (Tuckerman, 2010):

$$\begin{array}{l} Q=\mathrm{Tr}\left[e^{-\beta \hat{H}}\right]\\ =\oint Dx\left(\tau \right)\times \exp\left\{-\frac{1}{\hbar }\int_{0}^{\beta \hbar }d\tau \cdot \left[\frac{m}{2}{\dot{x}}^{2}\left(\tau \right)+U\left(x\left(\tau \right)\right)\right]\right\}\\ \approx {\int \text{d}x_{1}\ldots \text{d}x_{P}\times \exp\left\{-\frac{1}{\hbar }\sum_{k=1}^{P}\left[\frac{mP}{2\beta \hbar }{\left(x_{k+1}-x_{k}\right)}^{2}+\frac{\beta \hbar }{P}U\left(x_{k}\right)\right]\right\}|}_{x_{P+1}=x_{P}}\\ \approx Q_{P} \end{array}$$ (1)

In here, $Q_{P}$ stands for the geometric part of the partition function of a classical cyclic ring polymer with P beads. U(x) is the PES employed in the simulation, computed using either force field models or ab initio approaches. Exact quantum Boltzmann distribution can be obtained by taking P→∞ . In practice, we use limited number (typically P equals to 32 or 64 in room temperature) of slices to represent the continuous path of x(τ) . The conjugate momentum of the bead positions $x_{k}$ can be introduced in a variety of ways, leading to different flavors of path-integral-based methods such as RPMD, PA-CMD (partially adiabatic-CMD) (Hone et al., 2006), and CMD etc. In particular, CMD coarse grains the intra-ring vibrational modes and focuses on the physical motion of the geometric center of the ring-polymer (i.e., the “centroid”):

$$\begin{array}{l} x_{c}=\frac{1}{\hbar \beta }\int_{0}^{\hbar \beta }d\tau \;x\left(\tau \right)\\ \approx \frac{1}{P}\sum_{k=1}^{P}x_{k} \end{array}$$ (2)

When the motion of the centroid and the intra-ring vibrations are decoupled, the centroid can be considered as moving on an adiabatic PMF (denoted as $U_{c}\left(x\right)$ ), with the effective quantum centroid force defined as:

$$\begin{array}{l} F_{c}\left(x_{0}\right)=-\frac{dU_{c}\left(x_{0}\right)}{dx_{0}}\\ =-\frac{\oint Dx\left(\tau \right)\delta \left(x_{c}-x_{0}\right)\exp\left\{-S/\hbar \right\}\left[-\frac{\text{d}U\left(x\left(\tau \right)\right)}{\text{d}x}\right]}{\oint Dx\left(\tau \right)\delta \left(x_{c}-x_{0}\right)\exp\left\{-S/\hbar \right\}}\\ \approx {\langle F\left(x_{k}\right)\rangle |}_{x_{c}=x_{0}} \end{array}$$ (3)

Here, the action S is simply:

$$S=\int_{0}^{\beta \hbar }d\tau \cdot \left[\frac{m}{2}{\dot{x}}^{2}\left(\tau \right)+U\left(x\left(\tau \right)\right)\right]$$ (4)

In principle, one can conduct either MD or MC to sample the bead ( $x_{k}$ ) distributions while keeping the centroid positions ( $x_{c}$ ) fixed, then find the adiabatic centroid force by averaging the forces on the beads. But such scheme is computationally inefficient. Therefore, a common practice is to propagate all the motions simultaneously, but to set the masses of the intra-ring vibrations to be lighter, so these motions are decoupled from the physical centroid motion. However, such scheme requires a much smaller time step compared to regular classical MD simulations, thus causing extra computational cost.

Another simple alternative was proposed by Feynman and Hibbs (Feynman, 1998), when they proved that in a variational sense, the optimal effective classical potential K(x) that accounts for NQE is a Gaussian convolution of the underlying physical potential U(x) :

$$K\left(x\right)=\sqrt{\frac{6mkT}{\pi \hbar^{2}}}\int dx^{\prime }\cdot U\left(x^{\prime }\right)\exp\left[-\frac{6mkT{\left(x-x^{\prime }\right)}^{2}}{\hbar^{2}}\right]$$ (5)

It is interesting to observe that the Gaussian kernel features a width of $\sigma =\sqrt{\hbar^{2}/12mkT}$ , which happens to be the width of the ring-polymer bead distribution in the free particle limit (i.e., U(x)=0 ). Therefore, FHC can be viewed as an approximation to CMD when the shape of U does not affect the shape of the ring-polymer significantly.

When the underlying potential is pairwise additive and spherically symmetric (i.e., $U=\sum_{i<j}u\left(r_{ij}\right)$ ), Eq. 5 leads to a quite simple correction to the original pairwise interactions. This correction, truncated at second order, can be written as:

$$u^{FH}\left(r_{ij}\right)=u\left(r_{ij}\right)+\frac{\beta \hbar^{2}}{24\mu }\left[\frac{\partial^{2}u\left(r_{ij}\right)}{\partial r_{ij}^{2}}+\frac{2}{r_{ij}}\frac{\partial u\left(r_{ij}\right)}{\partial r_{ij}}\right]$$ (6)

Here, $\mu =\frac{1}{1/m_{i}+1/m_{j}}$ represents the reduced mass of the two interacting atoms. FHC provides an extremely simple solution to the NQE problem, avoiding the expensive explicit sampling of the ring polymer geometry. Therefore, FHC is widely used nowadays in complicated simulations such as the GCMC, which are often performed to compute the adsorption of gas molecules in porous materials. However, for a general many-body potential (e.g., ab initio PES), FHC does not give a simply analytical formula as given in Eq. 6, and cannot be easily computed without explicit MC sampling. Therefore, even though both CMD and FHC offer an effective quantum correction to the physical potential formally, neither of them can be conducted easily on a general many-body PES.

### Machine Learning Techniques and Model Locality

In this work, we will use the embedded atom neural network (EANN) technique to learn the quantum centroid force correction (Cao and Voth, 1994a; Feynman et al., 2010):

$$\Delta F_{c}\left(x_{0}\right)=F_{c}\left(x_{0}\right)-F\left(x_{0}\right)$$ (7)

EANN, like many other popular ML methods, assumes that the total energy (in this work, the centroid PMF) can be written as a sum of atomic energies:

$$U=\sum_{a}u_{a}\left({\vec{f}}_{a}\right)$$ (8)

And each atomic energy $u_{a}$ is a function of its local environment, the information of which is encoded in an atomic feature vector ${\vec{f}}_{a}$ . Neural networks (NN) are then employed to fit the complicated many-body function $u_{a}\left({\vec{f}}_{a}\right)$ . Different ML methods differ in the ways of constructing ${\vec{f}}_{a}$ , but the basic mathematical structure showed in Eq. 8 is always retained. One limitation of this scheme is that ${\vec{f}}_{a}$ can only encode the local atomic environment within a certain range $r_{c}$ , and the computational cost of the ML model increases rapidly with increasing $r_{c}$ . Many deficiencies of the ML methods manifested in the molecular force field development problem root in this limitation. Therefore, the ML technique is in natural favor of extremely localized problems, which require smaller distance cutoffs, thus resulting in more efficient training processes and faster computational speeds.

In this work, we will show that in molecular systems, the quantum centroid force correction ( $\Delta F_{c}$ ) happens to be an extremely localized target, so ML is a natural tool to learn this target. At first glance, it may not be very obvious why the machine learned quantum force correction is more efficient than the plain CMD. The training data collection could be painful: we need to fix the centroids at different geometries, and for each geometry, we have to run a thorough PIMD or PIMC simulation to fully converge the centroid force. This fully adiabatic procedure has been shown to be less efficient than the state-of-the-art CMD algorithm, which runs the ring-polymer dynamics on-the-fly using extended Lagrangian methods. However, in molecular systems, if the quantum force correction is localized enough, we can train the correction on small molecule clusters and apply it to bulk simulations. Such an approach brings a huge gain on computational efficiency, especially for potentials that are expensive to evaluate. Multipolar polarizable force fields (such as AMOEBA) and ab initio potentials are two typical examples: In the former case, bulk calculations involve the time-consuming multipolar Ewald summation, while cluster calculations with a simple cutoff scheme are much faster to run. In the latter case, the computational cost of regular DFT calculations scales as $O\left(N^{3}\right)$ , thus the bulk PIMD is also much harder to perform compared to small cluster sampling. Therefore, with the development of the new generation of accurate many-body force fields, it is becoming increasingly beneficial to train the NQE correction on small molecular clusters while using it in bulk simulations.

### Computational Details

In this work, we use water as our test example, with both q-TIP4P/F (Habershon et al., 2009) and AMOEBA (Ren and Ponder, 2003) force fields. The two force fields were selected due to the following reasons: 1) Both of them are not too expensive to run, so we can obtain the rigorous bulk PIMD benchmark data at a reasonable cost; 2) The two force fields represent two distinct categories of PES. The q-TIP4P/F force field is one of the conventional force fields with simple point charges and Lennard-Jones interactions, which are completely spherical symmetric and pairwise-additive. Meanwhile, AMOEBA features multipole moments and explicitly polarizable atoms, thus it resembles a more general many-body PES. Therefore, it is interesting to compare the performances of the localized ML model on both cases, in order to examine the generality of the method.

For each force field, we first performed a bulk (1,000 water) NPT PIMD simulation at the corresponding condition (1 bar, 300 K or 100 K), and randomly drew 2000 cluster configurations from the bulk trajectory. Each cluster was consisted by one central water molecule and seven nearest molecules, so we guarantee that the first solvation shell of the central water was fully included. These water octamers were then used to generate the training data: with centroid positions fixed, we ran 1 ns PIMD simulation to sample the bead distribution, so the averaged centroid force is converged within 0.2 kJ/(mol Å). To examine the width of the ring-polymer, we also performed 1 ns PIMD simulations on a smaller bulk system (with 216 water) with a density of 0.997 g/ml at different temperatures. All the sampling simulations mentioned above were conducted using the OpenMM 7.4.0 program, with modifications made to enable the fixed-centroid PIMD simulations. All sampling simulations were performed using 0.5 fs timestep and a Langevin thermostat with a 1.0 ps^−1^ friction constant. We used 32 beads at 300 K and 64 beads at 100 K, and the cutoff distance for the nonbonded interaction was set to 9 Å.

Once the averaged centroid forces were collected, their differences with the classical forces (i.e., $\Delta F_{c}$ ) were used to train an EANN model. The maximum angular momentum in the EANN model was set to 2, and 11 Gaussian Type Orbitals (GTOs) were used in the radial dimension. We used two hidden layers in the network, with 20 neurons in each layer. All the parameters in EANN were trained using the hybrid extreme machine learning and Levenberg-Marquardt (ELM-LM) algorithm, with the convergence criterion for the loss function set to be 0.1.

The trained EANN model was then used in combination with the original force field to perform the machine learning CMD (ML-CMD) simulations. For validation, the simulation results were compared to the PIMD, RPMD, and the PA-CMD results, and all the production runs were conducted using the i-PI 2.2 program. In PA-CMD simulations, the lower limit of internal modes is set to 648 THz to ensure the adiabaticity between the centroid mode and the intra-ring modes, and the timestep was set to be 0.02 fs.

## Results

### Locality of the Quantum Force Correction

As we discussed in the theory section, the locality of the $\Delta F_{c}$ is the key to the success of the ML models, and thus the key to the success of this work. Note that the quantum force correction is due to the smearing of the atom into a cyclic ring-polymer, so the correction would vanish if the size of the ring-polymer is small enough compared to the interaction range. Therefore, it is both interesting and necessary to rigorously quantify the distribution width of the ring-polymer at different conditions. Starting from Eq. 1, without losing generality, we assume that the centroid is located at $x_{c}=0$ , then expand and truncate the potential energy U around $x_{c}$ at the second order:

$$Q\approx \int Dx\left(\tau \right)\exp\left\{-\frac{1}{\hbar }\int_{0}^{\beta \hbar }d\tau \cdot \left[\frac{m}{2}{\dot{x}}^{2}+\frac{1}{2}U^{\prime \prime }x^{2}\right]\right\}$$ (9)

In here, $U^{\prime \prime }=\frac{\partial^{2}U}{\partial x^{2}}$ is the force constant of the potential at point $x_{c}$ along dimension x . Fourier transforming x (the n=0 term is dropped since we assume that $x_{c}=0$ ):

$$\left\{ \begin{array}{l} x\left(\tau \right)=\sum_{\underset{n\neq 0}{n=-\infty }}^{\infty }e^{i\omega_{n}\tau }x_{n}\\ \omega_{n}=\frac{2\pi n}{\beta \hbar }\;\left(n=\ldots ,-3,-2,-1,1,2,3,\ldots \right) \end{array}\right.$$ (10)

and we can rewrite Eq. 9 as:

$$Q=\int Dx\left(\tau \right)\exp\left\{-\frac{\beta }{2}\sum_{n}\left[m\omega_{n}^{2}+U^{\prime \prime }\right]{\left|x_{n}\right|}^{2}\right\}$$ (11)

Therefore, the distribution of $x_{n}$ is merely independent Gaussian distributions with the second moments:

$$\langle x_{n}^{2}\rangle =\frac{1}{\beta \left[m\omega_{n}^{2}+U^{\prime \prime }\right]}=\frac{1}{m\beta \left[\omega_{n}^{2}+\omega^{2}\right]}$$ (12)

And in here, the intrinsic vibrational frequency of the atom is: $\omega =\sqrt{\frac{U^{\prime \prime }}{m}}$ .

Therefore, the total second moment of the distribution of x can also be written as a sum of all Matsubara frequencies:

$$\langle x^{2}\left(\tau \right)\rangle =\overset{\infty }{\underset{\begin{array}{l} n=-\infty \\ n\neq 0 \end{array}}{\sum }}\langle x_{n}^{2}\rangle =\frac{1}{m\beta }\overset{\infty }{\underset{\begin{array}{l} n=-\infty \\ n\neq 0 \end{array}}{\sum }}\frac{1}{\omega_{n}^{2}+\omega^{2}}$$ (13)

Define the dimensionless quantity t as:

$$t=\frac{\beta \hbar \omega }{2\pi }$$ (14)

Then the second moment of the ring-polymer bead distribution is:

$$\begin{array}{l} \langle x^{2}\rangle =\frac{\beta \hbar^{2}}{4\pi^{2}m}\sum_{\underset{n\neq 0}{n=-\infty }}^{\infty }\frac{1}{n^{2}+t^{2}}\\ =\frac{\beta \hbar^{2}}{2\pi^{2}m}\left[-\frac{1}{2t^{2}}+\frac{\pi }{2t}\coth\left(\pi t\right)\right] \end{array}$$ (15)

Apparently, the locality of the ring-polymer is determined by both the temperature and the curvature of the underlying potential. In the high temperature limit ( β→0, t→0 ), Eq. 15 approaches the limit of $\frac{\hbar^{2}}{12mkT}$ , which is exactly the width used in the convolution kernel of FHC. When the temperature is high, the quantum effect is weak, so the ring-polymer is more similar to a classical particle with a very narrow width. In this case, the potential is smooth enough in the relevant neighborhood of the centroid, with the effects of its curvature being negligible. Therefore, the shape of the ring-polymer can be approximated well assuming the free-particle limit, and FHC can be viewed as an excellent approximation to CMD in the high-temperature region. However, in low temperature region, FHC fails miserably as it predicts a singular behavior with the distribution width going to infinitely large. Meanwhile, Eq. 15 in the low temperature limit ( β→∞, t→∞ ) gives a asymptotic value of $\langle x^{2}\rangle =\frac{\hbar }{2m\omega }$ , which is exactly the distribution width of the ground state of a quantum harmonic oscillator (QHO). This is also not surprising, since the particle should only populate the vibrational ground state in the low temperature limit. It can be further proved that both the QHO and the FHC widths are rigorously the upper bounds of the actual distribution width. The most delocalized (thus the most problematic) dimension is the one that involves the lowest frequency mode of the lightest atom.

**FIGURE 1:**
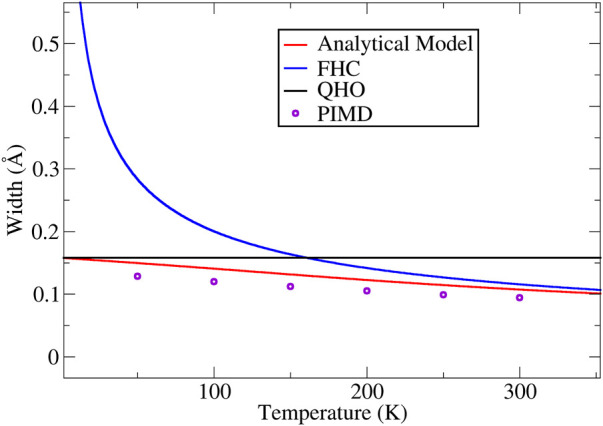
The width of the ring-polymer predicted by Eq. 15 at different temperatures (calculated using = 670 cm^−1^), plotted along the side with the FHC and QHO widths. The PIMD simulation results are also plotted in the figure using open circles.

In Figure 1, using liquid water as example, we plot the widths of the H atoms given by the square root of Eq. 15, in conjunction with the values given by the FHC and the QHO formula, as well as the PIMD simulation results. The intrinsic frequency ω is set to be 670 cm^−1^, which is approximately the frequency of the librational mode (i.e., the lowest frequency mode) of H in liquid water and ice (Tong et al., 2016). As it is shown, the analytical results agree with the simulation results in the entire temperature range, validating the utilization of Eq. 15 in the estimation of the bead distribution width. Numerically, bounded by both the QHO and the FHC limits, the average width of the H atoms is below 0.15 Å at all temperatures, showing that the PIMD bead distribution in a typical condensed molecular system is indeed extremely localized.

Using the results of the bead distribution width, we can roughly estimate the magnitude of the quantum force correction at a certain range. Let us consider a pair of particles interacting with Coulombic force (the force with the slowest decay rate in molecular systems), and evaluate how the quantum correction to the magnitude of the force decays with increasing distance. For convenience, without writing down the charges explicitly, we assume that the magnitude of the classical force between the two particles is:

$$F\propto \frac{1}{R^{2}}$$ (16)

And let us assume the three-dimensional ring-polymer bead shifts for particle *i* and particle *j* are ${\vec{x}}_{i}$ and ${\vec{x}}_{j}$ , respectively. Both ${\vec{x}}_{i}$ and ${\vec{x}}_{j}$ are with respect the centroids, thus we have: $\langle {\vec{x}}_{i}\rangle =\langle {\vec{x}}_{j}\rangle =0$ .

Then, the averaged quantum force correction, truncated at second order, is:

$$\begin{array}{l} \Delta F=\langle \frac{1}{{\left|\vec{R}+{\vec{x}}_{i}-{\vec{x}}_{j}\right|}^{2}}\rangle -\frac{1}{R^{2}}=\langle \frac{1}{{\left|\vec{R}+\Delta \vec{x}\right|}^{2}}\rangle -\frac{1}{R^{2}}\\ \approx \langle \Delta \vec{x}\cdot \left[\frac{4\left(\vec{R}\vec{R}\right)-R^{2}I}{R^{6}}\right]\cdot \Delta \vec{x}\rangle \\ =\frac{1}{R^{4}}\left(4\langle \left(\vec{n}\cdot \Delta \vec{x}\right)\left(\vec{n}\cdot \Delta \vec{x}\right)\rangle -\langle \Delta \vec{x}\cdot \Delta \vec{x}\rangle \right) \end{array}$$ (17)

Hence the relative correction is:

$$\frac{\Delta F}{F}\approx \frac{1}{R^{2}}\left(4\langle \left(\vec{n}\cdot \Delta \vec{x}\right)\left(\vec{n}\cdot \Delta \vec{x}\right)\rangle -\langle \Delta \vec{x}\cdot \Delta \vec{x}\rangle \right)$$ (18)

Considering $\Delta x=\vec{n}\cdot \Delta \vec{x}$ is simply the one-dimensional projection of $\Delta \vec{x}$ along the direction of $\vec{R}$ , and $\langle \Delta \vec{x}\cdot \Delta \vec{x}\rangle \geq \langle \Delta x^{2}\rangle$ , we have:

$$\begin{array}{l} \frac{\Delta F}{F}\leq \frac{1}{R^{2}}\left(4\langle \Delta x^{2}\rangle -\langle \Delta x^{2}\rangle \right)\\ =\frac{3}{R^{2}}\left[\langle x_{i}^{2}\rangle +\langle x_{j}^{2}\rangle -2\langle x_{i}x_{j}\rangle \right] \end{array}$$ (19)

The largest force correction happens when the direction of $\vec{R}$ (i.e., $\vec{n}$ ) coincides with the lowest frequency mode (the librational mode) of hydrogen. Therefore, we can simply use the librational frequency and Eq. 15 to evaluate the upper bounds for $\langle x_{i}^{2}\rangle$ and $\langle x_{j}^{2}\rangle$ . According to Cauchy-Schwarz inequality, we further have: $\left|\langle x_{i}x_{j}\rangle \right|\leq \sqrt{\langle x_{i}^{2}\rangle \langle x_{j}^{2}\rangle }=\langle x^{2}\rangle$ . Therefore, we have:

$$\frac{\Delta F}{F}\leq \frac{12}{R^{2}}\langle x^{2}\rangle$$ (20)

This inequality could be further improved, realizing that the correlation ( $\langle x_{i}x_{j}\rangle$ ) also decays as $1/R^{3}$ (see supporting information for more analysis to the position and force correlations). However, Eq. 20 is already good enough for the following numerical analysis.

In this work, we use a cutoff distance of 4 Å for our ML model, and at this distance, Eq. 20 gives a relative force correction as small as 2% at all temperatures. Furthermore, both analytical and numerical analysis indicate that the correlation between the force corrections decays fast with respect to interatomic distance. Therefore, it is strongly indicated that the quantum force correction is an extremely localized phenomenon and can be tackled using a very small localized ML model.

It is noted that previous studies (Rognoni et al., 2021) show the vibrational modes of liquid water is highly nonlocal. While there are certainly nonlocal NQE existing in aqueous systems, such result is not necessarily contradictory to our findings in this work. In here, we focus on the locality of the centroid force correction: just like localized force can lead to nonlocal vibrational modes, local force correction can certainly give rise to global NQE in bulk systems.

### Force Tests

**FIGURE 2:**
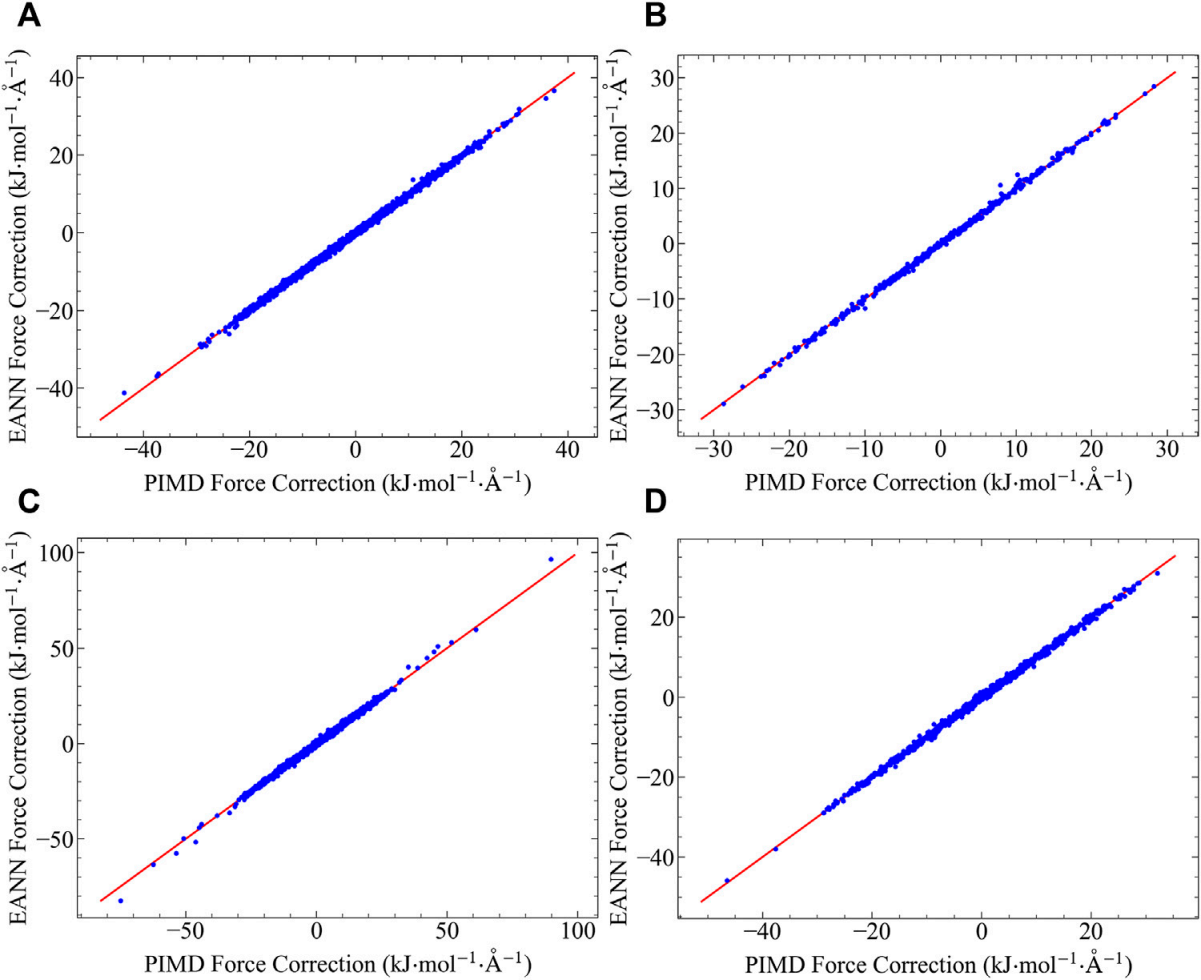
Quantum force corrections predicted by the EANN model, plotted against the reference data computed using PIMD: **(A)** Results in bulk water with q-TIP4P/F force field. **(B)** Results in surface water with q-TIP4P/F force field. **(C)** Results in bulk water with AMOEBA force field. **(D)** Results in surface water with AMOEBA force field.

Using the water octamer PIMD centroid forces as training data, we train the EANN model with a small cutoff of 4 Å, and test its validity in both bulk and surface water systems. We examine the accuracy of the EANN model on the quantum force corrections for both Q-TIP4P/F and AMOEBA potentials, and the test results are shown in Figure 2. Excellent agreement between the fitted and the reference data are achieved in all cases, showing the great success of the methodology. This result is in consistent with our analysis in the last section, showing that the quantum force correction is virtually fully determined by the structure of the first solvation shell. Water octamer clusters that contain one fully solvated water molecule and seven surface molecules is all it needs to obtain a model that is transferrable to both bulk and surface environments. Such extrapolation capability does not rely on the pairwise additivity of the underlying potential, and is extremely important for PES that is difficult to evaluate (such as AMEOBA).

### Thermodynamic Properties

**FIGURE 3:**
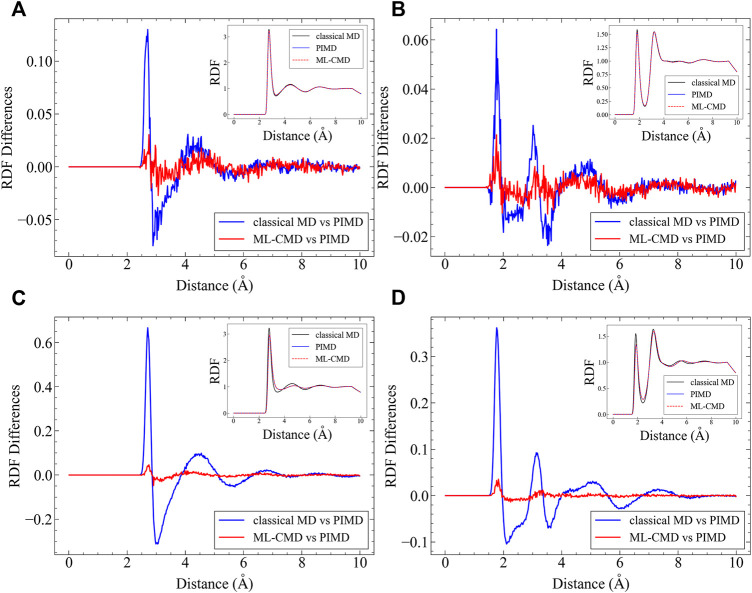
Comparison between different centroid RDFs. In each figure, the RDF differences between ML-CMD and PIMD are shown, in conjunction with the RDF differences between classical MD and PIMD. The corresponding RDFs are all plotted in the inset. **(A)** The O-O RDF in q-TIP4P/F simulations; **(B)** the O-H RDF in Sq-TIP4P/F simulations; **(C)** the O-O RDF in AMOEBA simulations; and **(D)** the O-H RDF in AMOEBA simulations.

After examining the centroid force correction, we investigate the performance of the EANN model in the bulk ML-CMD simulations. We first investigate the centroid radial distribution functions (RDFs) for both O-O and O-H atom pairs, and the results are plotted in Figure 3. It is noted that the RDF reported here is not the regular RDF reported in literature, but the RDF computed by centroid positions. Although in principle, there is no essential technical difficulties to train the PMF of one bead (instead of the centroid) such that the real RDF can be obtained. Apparently, compared to pure classical MD, the machine learned quantum force correction reduces the error on the centroid RDFs significantly compared to the exact PIMD simulation. It is further observed that the NQE on the centroid RDFs are more significant in the AMOEBA simulations compared to the q-TIP4P/F simulations, and our correction is also more effective in AMOEBA. For both force fields, the quantum centroid RDF is generally less structured compared to the classical results, and such effect is accurately captured by the EANN model.

**FIGURE 4:**
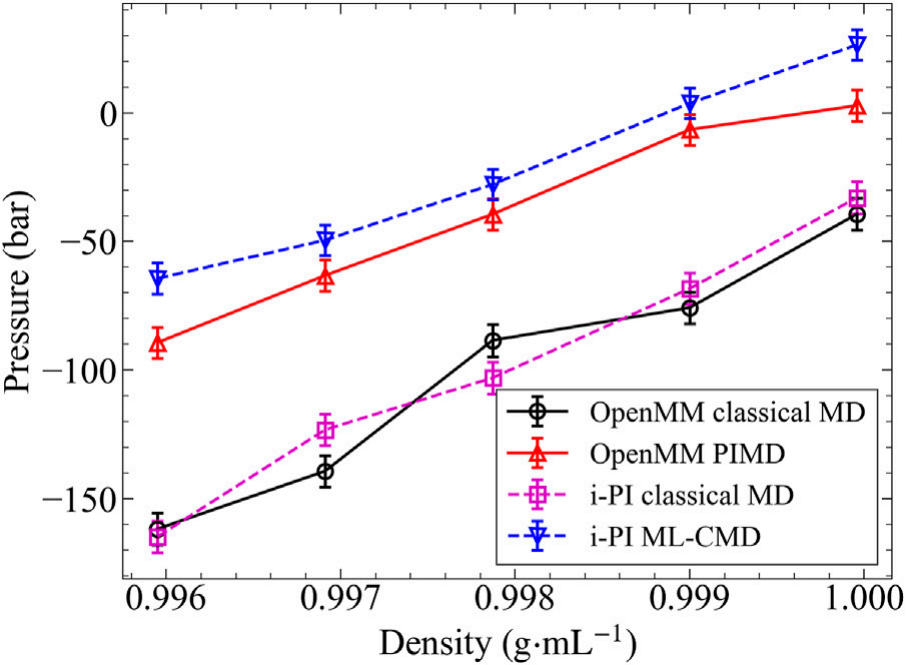
The pressure varies with density in 300 K. This is an alternative pressure plot because the barostat behave difference in two different software. The black solid line: classical MD simulated by OpenMM. The violet dashed line: classical MD simulated by i-PI. The red solid line: PIMD simulated by OpenMM. The dashed blue line: ML-CMD simulated by i-PI This model is trained in 0.998 g/ml.

Besides the liquid structure, we further examine the NQEs on the system pressure (or, equivalently, the system density), and the results are shown in Figure 4. In this part, we performed the reference PIMD simulations using the more efficient OpenMM program, while the ML-CMD was conducted using i-PI for convenience. To rule out the potential numerical differences caused by different programs, we computed the classical baselines using both codes. The classical pressures computed by both OpenMM and i-PI are in excellent agreement, proving that the different integrators and thermostat implementations in the two codes do not affect our simulation results significantly. With the classical baselines established, it is clearly shown in Figure 4 that ML-CMD reproduces the exact PIMD results with a reasonable accuracy. Small overestimations can be observed in both the low-density and the high-density ends, potentially because that the EANN model was trained at 0.998 g/ml, so the model error is slightly larger when the density deviates from this density. In summary, it is shown that in both the centroid RDF and the pressure tests, the ML force correction trained by only small cluster data can reproduce the bulk PIMD results accurately, proving the capability of our methodology in the studies of thermodynamic properties.

### Dynamic Property

As we discussed in the theoretical background section, the ML-CMD method is theoretically equivalent to adiabatic CMD, so it should also be able to capture the NQE in dynamic properties at CMD level of theory. We thus computed the self-diffusion constant of bulk water using classical MD, PA-CMD, and ML-CMD, the results of which are listed in Table 1. The effective mass used in PA-CMD is small enough such that the PA-CMD simulation we perform is a good approximation to the rigorous adiabatic CMD. Once again, the ML-CMD agrees with PA-CMD within the statistical uncertainty, validating our methodology.

**TABLE 1:** The self-diffusion constants from classical MD, PA-CMD, and ML-CMD simulations, respectively. The last digit in the parenthesis marks the uncertainty.

	Classical MD	PA-CMD	ML-CMD
Diffusion constant (Å2/ps)	0.193 (4)	0.224 (5)	0.220 (4)

### Computational Cost

To verify that ML-CMD indeed possesses significant advantages in computational efficiency, we compare the running speeds of classical MD, T-RPMD, PA-CMD, and ML-CMD in Table 2. AMOEBA force field is employed, and all simulations are run using i-PI, with one RTX 2080 graphic card, 8 CPU cores, and 80 GB of memory. The EANN model has not been implemented in CUDA platform, so this part of the calculation is performed on a separate CPU client, while all other calculations are run on GPU. Comparing ML-CMD with classical MD, we can see that even with a slow CPU implementation, the extra computational time due the EANN model is minor. Because of the extremely short cutoff distance (4 Å) we use, the EANN model is rather small and is very fast to evaluate. Meanwhile, both T-RPMD and PA-CMD are orders of magnitude slower than ML-CMD and classical MD, due to two major reasons. First, both simulations have to propagate the motions of 32 replicas of the system, which means at least 32 times slower due to more energy and force evaluations. Second, the timestep has to be smaller in both T-RPMD and CMD to maintain the stability of the simulations. We do acknowledge that a better integrator can be employed so a larger timestep is possible for at least T-RPMD, and techniques such as contraction can help to decrease the number of beads employed in force evaluation. All these techniques can alleviate, but not cure the heavy computational cost of path integral simulations. In comparison, ML-CMD is by far the simplest, and the most straightforward way to achieve the classical-like simulation speed, circumventing the complicated and highly specialized techniques used in PIMD.

**TABLE 2:** The simulate resources and time costs of different methods.

	Classical MD	T-RPMD	PA-CMD	ML-CMD
Clients	1 on GPU	32 on GPU	32 on GPU	1 on GPU and 1 on CPU
GPU card	1	1	1	1
CPU cores	8	8	8	8
Memory (MB)	80,000	80,000	80,000	80,000
Steps	20,000	100,000	500,000	20,000
Step length (fs)	0.5	0.1	0.02	0.5
Simulation length (ps)	10	10	10	10
Time (h)	0.2	8	40	0.3

## Conclusion and Outlook

In this work, through both analytical and numerical approaches, we analyze the locality of the centroid force corrections in typical molecular systems such as bulk water. A general formula is given to quantify the locality, and it was found that at ambient condition, the NQE on the forces decays to less than 0.5% at the range of 4 Å. Exploiting this locality, we are able to train a reliable ML model to predict the NQE force correction, utilizing only small cluster training data. For a PES that is expensive to evaluate (e.g., polarizable force field or even ab initio potential), such locality is particularly important as the force correction in bulk system can be difficult, or even impossible to compute. Also due to the locality, the model is high transferrable to different physical environments, and applicable to both pairwise additive force fields and general many-body potentials. The ML model is formulated as a simple correction term to the classical forces and energies, thus is straightforward to implement and easy to use in complicated simulations such as GCMC. The ML-CMD simulation, which uses the ML quantum force corrections in combination with the classical forces, reproduces both the thermodynamics and the dynamics of adiabatic CMD. Thanks to the extreme locality of the model, the ML-CMD simulation runs as efficient as classical MD, which is much faster compared to both RPMD and CMD. The computational cost of ML-CMD is not significantly higher than FHC, while ML-CMD is formulated more rigorously and is more accurate compare to FHC. Therefore, the localized ML-CMD is considered to be an excellent approach to incorporate NQEs in the simulations of large biological and materials systems.

Meanwhile, we note that there are a few scenarios that the locality of the ML-CMD could be broken. One typical example is the proton tunneling under low temperature: Eq. 15 holds only when the curvature of the underlying PES is positive, which is not the case in the transition state region of a chemical reaction. At high temperature, the locality is still enforced by the FHC limit, but in low temperature, the locality of the proton at the transition region could be a problem. More detailed analysis is needed to quantify the necessary cutoff range of the ML model in these cases, which is left to future work. Nevertheless, in most nonreactive molecular system simulations (e.g., protein folding, drug molecule binding etc.), our method serves as a both robust and fast alternative to the rigorous PIMD and adiabatic CMD simulations.

## Data Availability

The raw data supporting the conclusion of this article will be made available by the authors, without undue reservation.
