# Supplementary material for: Learning the Quantum Centroid Force Correction in Molecular Systems: A Localized Approach
Source: Front Mol Biosci. 2022 May 19;9:851311. doi: 10.3389/fmolb.2022.851311 (PMC9161153; doi:10.3389/fmolb.2022.851311)
Supplement: Supplementary file 1 [file DataSheet1.docx]

Supporting Information for “Learning the Quantum Centroid Force Correction in Molecular Systems: A Localized Approach”

Chuixiong Wu^1†^, Ruye Li^1†^, and Kuang Yu^1*^

^1^ Tsinghua-Berkeley Shenzhen Institute (TBSI), Tsinghua Shenzhen International Graduate School, Tsinghua University, 1101 Xueyuan Road, Building C2, Shenzhen, Guangdong, 518055, China

**^†^** These authors share first authorship

*** Correspondence:**Corresponding Author
[yu.kuang@sz.tsinghua.edu.cn](mailto:yu.kuang@sz.tsinghua.edu.cn)

# Correlation Between Bead Positions and Bead Forces

We first start our analysis from the partition function of a many-body system. For simplicity, we assume all particles have the same mass $m$. If we expand the potential energy surface around the centroid position and truncate at second order, we have:

And $H_{ij}=\frac{\partial U}{\partial x_{i}\partial x_{j}}$ is the Hessian matrix of the entire system, and index $i$ runs over all dimensions (x, y, and z) of all atoms. Again, expand $x(\tau)$ as sum of Matsubara frequencies:

Then,

In here, $\bar{H}=\frac{H}{m}$ is the mass-weighted Hessian matrix. This is a standard multivariate Gaussian distribution, thus we can obtain the correlation for $x_{n}^{i}$:

$$\left\langle x_{n}^{i}x_{n}^{j}\right\rangle=\frac{kT}{m}\left(\omega_{n}^{2}I+\bar{H}\right)_{ij}^{-1}$$

Then the correlation in real space can be written as:

$$\begin{matrix} \left\langle x_{i}x_{j}\right\rangle=\sum_{n\neq0} \left\langle x_{n}^{i}x_{n}^{j}\right\rangle\\ =\sum_{n\neq0} \frac{kT}{m}\left(\omega_{n}^{2}I+\bar{H}\right)_{ij}^{-1} \end{matrix}$$

It is difficult to derive a simple analytical result from Eqn. due to the matrix inversion involved in the formula. However, we will proceed by decomposing $\bar{H}$ into two parts: the intramolecular part ($\bar{H}_{0}$) and the intermolecular part ($\bar{H}_{1}$):

$$\bar{H}=\bar{H}_{0}+ \bar{H}_{1}$$

Then Eqn. can be transformed to:

$$\begin{matrix} \left\langle x_{i}x_{j}\right\rangle=\sum_{n\neq0} \frac{kT}{m}\left(\omega_{n}^{2}I+\bar{H}_{0}+\bar{H}_{1}\right)_{ij}^{-1} \\ =\sum_{n\neq0} \frac{kT}{m}\left(A+\bar{H}_{1}\right)_{ij}^{-1} \end{matrix}$$

With:

$$A=\omega_{n}^{2}I+\bar{H}_{0}$$

Here, matrix $A$ is rigorously localized: in the case of water, it is blockwise diagonal, so does its inversion $A^{-1}$. The Intermolecular Hessian $\bar{H}_{1}$ is not diagonal, but it can be argued that its magnitude is much smaller compare to $A$ (as the intermolecular potential is considered to be much “softer” compared to intramolecular interactions). Therefore, we will treat $\bar{H}_{1}$ as a perturbation to $A$. Using the fact that:

$$(A+\lambda B)^{-1}=A^{-1}+ \lambda A^{-1}BA^{-1}+O(\lambda^{2})$$

We can approximate Eqn. as:

$$\left\langle x_{i}x_{j}\right\rangle==\sum_{n\neq0} \frac{kT}{m}\left(A^{-1}+A^{-1}\bar{H}_{1}A^{-1}+\ldots\right)_{ij}$$

Here, as we discussed, all the long-range off-diagonal elements come from the second term ($A^{-1}\bar{H}_{1}A^{-1}$). While $A^{-1}$is rigorously blockwise diagonal, it does not introduce nonlocal couplings. Therefore, for any $i$ and $j$ that are far apart, their correlations approximately scales with $H_{1}/m$. For the most long-ranged Coulombic interactions, we have $U\propto\frac{1}{R}$, thus:

$$H_{1}=\frac{\partial U}{\partial x_{1}\partial x_{2}}\propto\frac{1}{R^{3}}$$

Then we reach the conclusion that $\left\langle x_{i}x_{j}\right\rangle\propto\frac{1}{R^{3}}$. Therefore, the correlation between the bead positions also decays in a reasonably fast rate with respect to the interatomic distance.

In Figure 1, we show the numerical results of $\left\langle\vec{x}_{i}\cdot\vec{x}_{j}\right\rangle$ sampled using PIMD, and plotted against interatomic distance $R$ between $i$ and $j$. It is clear that the correlation drops to a really small value (<0.05) at the distance of 4.0 Å.


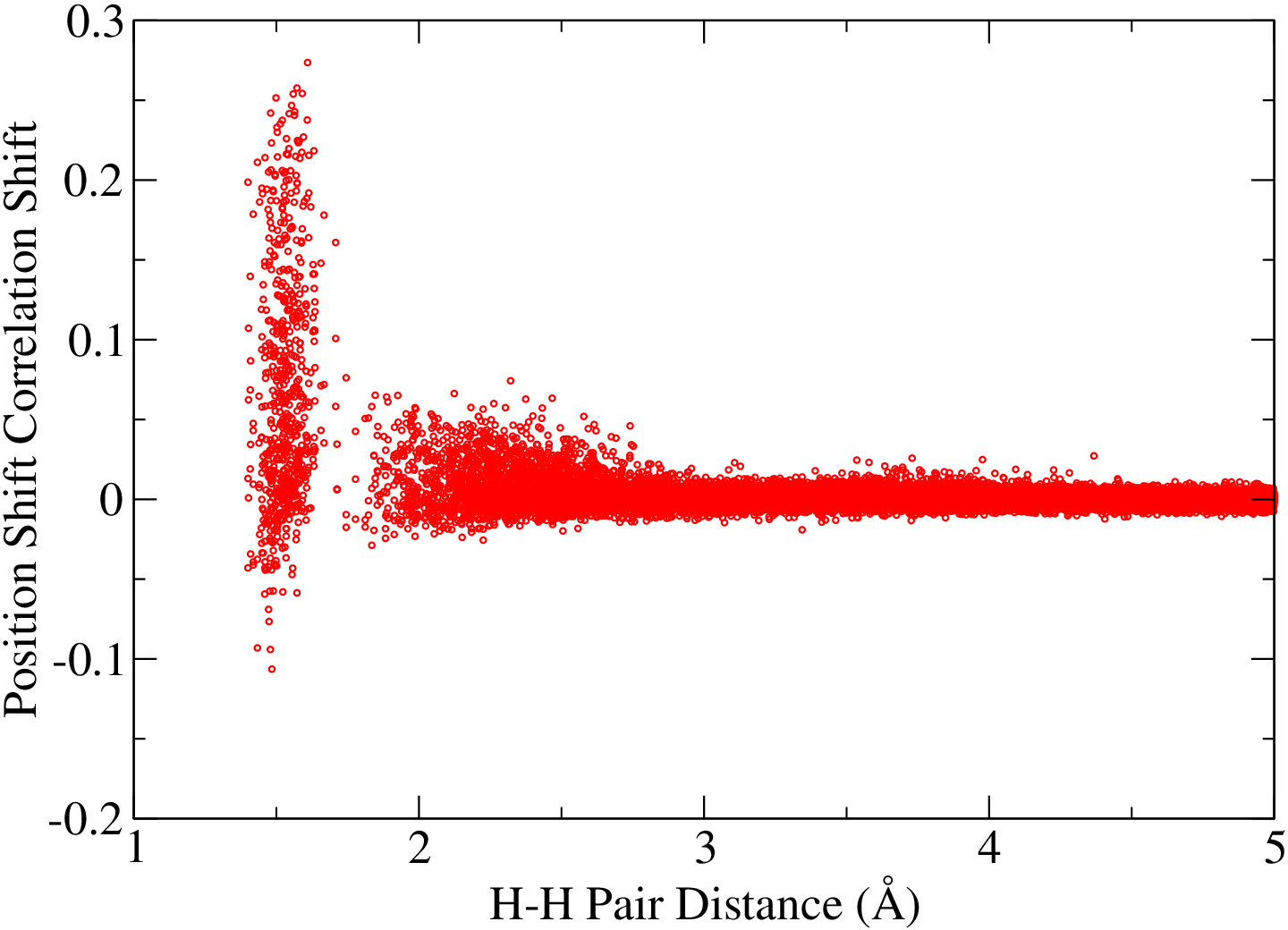


Figure . The correlation coefficients between the bead positions, plotted against the interatomic distances.

With the position correlation in hand, we can evaluate the correlation between the force corrections:

$$\left\langle\Delta F_{i}^{\alpha}\Delta F_{j}^{\beta}\right\rangle\approx\sum_{\gamma\delta} \left\langle H_{i}^{\alpha\gamma}x_{i}^{\gamma}H_{j}^{\beta\delta}x_{i}^{\delta}\right\rangle$$

In here, $H_{i}$ and $H_{j}$ is the local Hessian for particle $i$ and $j$, respectively. Then,

$$\left\langle\Delta\vec{F}_{i}\Delta\vec{F}_{j}\right\rangle\approx H_{i}\left\langle\vec{x}_{i}\vec{x}_{j}\right\rangle H_{j}\propto O\left(\frac{1}{R^{3}}\right)$$

The correlation of force linearly depends on the correlation of bead position, thus also decays with the same rate with respect to $R$. In Figure 2 we show the numerical result for force correlations, which clearly shows its fast decay behavior.


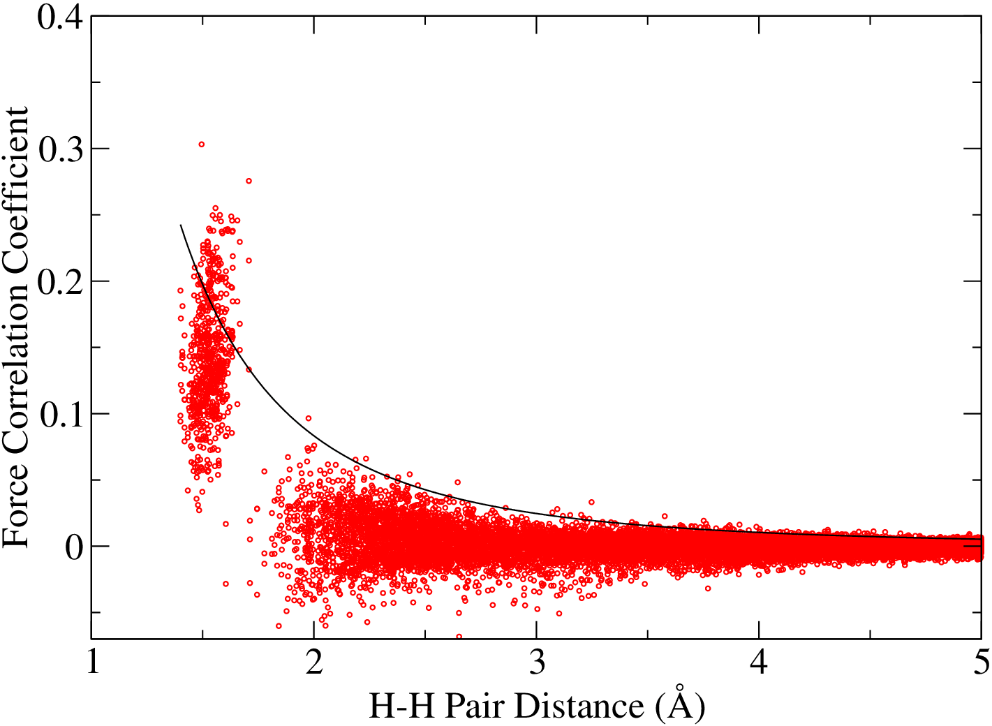


Figure . The correlation coefficients between the bead forces, plotted against the interatomic distances. A trend line is plotted that scales as $1/R^{3}$.
